# Neonatal Exposure to Brominated Flame Retardant BDE-47 Reduces Long-Term Potentiation and Postsynaptic Protein Levels in Mouse Hippocampus

**DOI:** 10.1289/ehp.9860

**Published:** 2007-02-05

**Authors:** Milou M.L. Dingemans, Geert M.J. Ramakers, Fabrizio Gardoni, Regina G.D.M. van Kleef, Åke Bergman, Monica Di Luca, Martin van den Berg, Remco H.S. Westerink, Henk P.M. Vijverberg

**Affiliations:** 1 Toxicology Division, Institute for Risk Assessment Sciences, Utrecht University, Utrecht, the Netherlands; 2 Rudolf Magnus Institute of Neuroscience, University Medical Centre, Department of Pharmacology and Anatomy, Utrecht, the Netherlands; 3 Department of Pharmacological Sciences and Center of Excellence on Neurodegenerative Diseases, University of Milan, Milan, Italy; 4 Department of Environmental Chemistry, Wallenberg Laboratory, Stockholm University, Stockholm, Sweden

**Keywords:** αCaMKII, brain growth spurt, developmental neurotoxicity, field-EPSP recording, hippocampal synaptic plasticity, postsynaptic density

## Abstract

**Background:**

Increasing environmental levels of brominated flame retardants raise concern about possible adverse effects, particularly through early developmental exposure.

**Objective:**

The objective of this research was to investigate neurodevelopmental mechanisms underlying previously observed behavioral impairments observed after neonatal exposure to polybrominated diphenyl ethers (PBDEs).

**Methods:**

C57Bl/6 mice received a single oral dose of 2,2′,4,4′-tetrabromodiphenyl ether (BDE-47) on postnatal day (PND) 10 (i.e., during the brain growth spurt). On PND17–19, effects on synaptic plasticity, levels of postsynaptic proteins involved in long-term potentiation (LTP), and vesicular release mechanisms were studied *ex vivo*. We investigated possible acute *in vitro* effects of BDE-47 on vesicular catecholamine release and intracellular Ca^2+^ in rat pheochromocytoma (PC12) cells.

**Results:**

Field-excitatory postsynaptic potential (f-EPSP) recordings in the hippocampal CA1 area demonstrated reduced LTP after exposure to 6.8 mg (14 μmol)/kg body weight (bw) BDE-47, whereas paired-pulse facilitation was not affected. Western blotting of proteins in the postsynaptic, triton-insoluble fraction of hippocampal tissue revealed a reduction of glutamate receptor subunits NR2B and GluR1 and autophosphorylated-active Ca^2+^/calmodulin-dependent protein kinase II (αCaMKII), whereas other proteins tested appeared unaffected. Amperometric recordings in chromaffin cells from mice exposed to 68 mg (140 μmol)/kg bw BDE-47 did not reveal changes in catecholamine release parameters. Modest effects on vesicular release and intracellular Ca^2+^ in PC12 cells were seen following acute exposure to 20 μM BDE-47. The combined results suggest a post-synaptic mechanism *in vivo*.

**Conclusion:**

Early neonatal exposure to a single high dose of BDE-47 causes a reduction of LTP together with changes in postsynaptic proteins involved in synaptic plasticity in the mouse hippocampus.

Fetal and neonatal exposure to neurotoxicants have adverse effects on neurodevelopment. Early (small) effects of xenobiotics on the brain could aggravate these effects during development, creating a critical window for neurotoxicity. However, the underlying mechanisms are not well understood (Szpir 2006). Recently, a range of behavioral and neurochemical effects have been described for polychlorinated biphenyls (PCBs) (for review, see Fonnum et al. 2006; Mariussen and Fonnum 2006). Nowadays, the increasing concentrations of the structurally related polybrominated diphenyl ethers (PBDEs) in the environment, human food chain, and human tissues (Hites 2004) raise concern about possible neurotoxic effects. In most samples, 2,2′,4,4′-tetrabromodiphenyl ether (BDE-47) is the predominant congener. PBDEs are used as flame retardants in a range of products, including electronic equipment, furniture, construction materials, and textiles.

Of concern is that children, at the age of early brain development, accumulate BDE-47 more rapidly than adults because of their diet (breast-feeding/relatively large intake) and behavior (contact with house-dust) (Jones-Otazu et al. 2005). Distribution studies show that developing mice reach higher tissue concentrations of BDE-47 compared with adult mice after identical dosing regimens (Staskal et al. 2006). Behavioral studies have demonstrated adverse neurodevelopmental effects on learning and memory after neonatal BDE-47 exposure. Habituation capability in mice, studied by scoring spontaneous behavior after placement in a new environment, is reduced and this effect is long-lasting and increases with age (Eriksson et al. 2001).

Recently, a proteomics approach was used to investigate the effect of a single oral dose of 12 mg (21.2 μmol)/kg body weight (bw) 2,2′,4,4′,5-pentabromodiphenyl ether (BDE-99) on brain protein levels in mice, 24 hr after exposure. Levels of striatal proteins associated with neurodegeneration and neuroplasticity and of hippocampal proteins associated with metabolism and energy production were found to be changed (Alm et al. 2006). It is unclear whether such changes occur after exposure to other congeners, and whether these protein changes have functional consequences.

The main objective of our study was to gain insight in the mechanisms underlying the observed effects of BDE-47 on learning and memory (Eriksson et al. 2001). To this purpose we have investigated *N*-methyl-d-aspartate (NMDA)-dependent long-term potentiation (LTP) in hippocampal slices from animals exposed to a dose of BDE-47 known to induce behavioral aberrations. NMDA-dependent LTP has been used as an electrophysiologic substrate for learning and memories for many years. This form of LTP is induced by tetanic stimulation, strong depolarization, and a large increase in intra-cellular Ca^2+^ level (for review, see Lynch 2004; Malenka and Nicoll 1999; Soderling and Derkach 2000). Paired pulse facilitation (PPF), a form of short-lasting plasticity that presumably reflects presynaptic function (Xu-Friedman and Regehr 2004), was investigated to reveal possible presynaptic effects of BDE-47. In additional *ex vivo* experiments, we investigated protein expression levels in the postsynaptic density (PSD) and catecholamine release from chromaffin cells to further reveal underlying mechanisms. Acute effects of BDE-47 on intracellular Ca^2+^ and catecholamine release of PC12 cells have been studied *in vitro* to assess the involvement of transient acute effects on potential presynaptic targets. Our findings provide a functional basis for previously observed neurobehavioral changes (Eriksson et al. 2001).

## Materials and Methods

### Animals and chemicals

Male C57Bl/6 mice pups (litters culled to 5 pups each) with mother (Harlan, Horst, the Netherlands) were housed in a standard animal facility on a 12-hr light/dark cycle with food and water *ad libitum*. Animals were treated humanely and with regard for alleviation of suffering. All experimental procedures were performed according to Dutch law and approved by the Ethical Committee for Animal Experimentation of Utrecht University.

Male C57Bl/6 mice received a single oral dose of vehicle [1:10 (wt/wt) mixture of egg lecithin (Sigma-Aldrich, Zwijndrecht, the Netherlands) and peanut oil (*Oleum arachidis*) (Sigma-Aldrich), sonicated with water to obtain a 20% (wt/wt) fat:water emulsion] or 6.8 mg (14 μmol)/kg bw BDE-47 via a metal gastric tube on postnatal day (PND) 10 for field-excitatory postsynaptic potential (f-EPSP) recordings and brain protein analysis, or 68 mg (140 μmol)/kg bw BDE-47 for amperometric recordings of chromaffin cells (to investigate presynaptic effects) at PND17–19. Experimental groups consisted of mice from different nests.

BDE-47 was synthesized and purified (~ 99%) at the Wallenberg laboratory of Stockholm University. For oral dosing, BDE-47 was dissolved in the egg lecithin/peanut oil mixture and sonicated with water to obtain a 20% (wt/wt) fat:water emulsion.

### Hippocampal slice preparation

On PND17–19 (directly after brain growth spurt), the animals were killed by decapitation after inhalation anesthesia (isoflurane), and the brain was rapidly dissected on ice. Hippocampal slices were prepared as described previously (Van der Heide et al. 2005). Briefly, transverse hippocampal slices (450 μm) were cut in ice-cold carbogenated Mg^2+^-enriched artificial cerebrospinal fluid (ACSF) [containing NaCl (124 mM), KCl (3.3 mM), KH_2_PO_4_ (1.2 mM), MgSO_4_ (2.6 mM), CaCl_2_ (2.5 mM), NaHCO_3_ (20 mM), and glucose (10 mM)] using a Leica VT1000 S vibrotome (Leica Microsystems, Wetzlar, Germany). The slices were allowed to stabilize at room temperature in carbogenated ACSF (MgSO_4_: 1.3 mM) for at least 1.5 hr.

### Extracellular recording of field potentials

**Figure 1:**
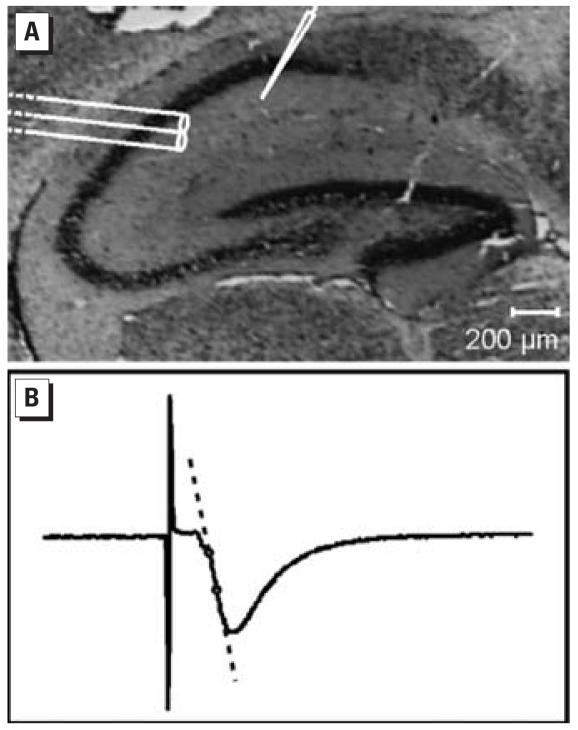
f-EPSP recordings in the CA1 region of hippocampal slices. (*A*) Placement of bipolar stimulation electrode and ACSF-filled microelectrode in CA1 region in a Nissl-stained hippocampal slice; bar = 200 μm. (*B*) Representative f-EPSP recording. For data analysis, the initial slope is determined between dots.

We recorded f-EPSPs in the CA1 region of hippocampal slices as previously described by Van der Heide et al. (2005), with minor modifications. Slices were superfused with carbogenated ACSF (~ 2 mL/min) in a recording chamber at 30°C. A bipolar stainless steel stimulation electrode (Ø 0.1 mm) was placed on the afferent fibers of the stratum radiatum of the hippocampal CA1 region, as shown in a Nissl-stained hippocampal slice in Figure 1A. f-EPSPs were recorded with ACSF-filled glass micro-electrodes using an Axoclamp-2B amplifier (Axon Instruments, Foster City, CA, USA). Data were digitized and stored using “Spike2” software (Cambridge Electronic Design, Cambridge, UK).

Stimulation intensities for threshold and maximum f-EPSPs were determined. Slices with a maximum response amplitude of ≥1 mV were included in the experiment. During baseline recording, half-maximum f-EPSPs were evoked every 30 sec. After 15 min baseline recording, LTP was induced with a single tetanic stimulation (100 Hz, 1 sec) and f-EPSPs were recorded for another 30 min. PPF, with interstimulus intervals of 50, 100, 200, 500, and 1,000 msec, was recorded under identical conditions as for LTP. For data analysis, we determined initial slopes of the f-EPSPs (Figure 1B).

For quantification of LTP, the slope was normalized against the average f-EPSP slope during baseline. Average relative increase of the slope was determined 20–30 min after tetanic stimulation as a measure for LTP and 0–7.5 min after tetanic stimulation as a measure for posttetanic potentiation (PTP) in the individual animals. To determine PPF, paired-pulse ratio (PPR) was determined by dividing the slope of the second average f-EPSP by the slope of the first average f-EPSP (*n* = 10).

### Western blotting analysis

We performed Western blotting analysis as described previously by Gardoni et al. (2006), with minor modifications. The triton-insoluble fraction (TIF) was purified from blind samples of single cortices and hippocampi of control (*n* = 4) and BDE-47–exposed animals [6.8 mg (14 μmol)/kg bw; *n* = 4] using a previously validated biochemical fractionating method (Gardoni et al. 2006), in the presence of protease inhibitors (CompleteTM; Roche Diagnostics, Basel, Switzerland) and phosphatase inhibitors (Sigma, St. Louis, MO, USA). Similar protein yield was obtained in TIF purified from cortex (~ 200 μg) and hippocampi (~ 50 μg) of both groups. Protein composition of this preparation was tested for the absence of presynaptic marker synaptophysin (Gardoni et al. 2001) and enrichment in the PSD proteins (Gardoni et al. 2006). Samples (3 μg) were applied to SDS-PAGE and electroblotted. For each TIF preparation three independent western blotting experiments were run. After blocking nonspecific protein interactions with 10% albumin in Tris-buffered saline (TBS), the nitrocellulose papers were incubated for 2 hr at room temperature with the primary antibodies: NR1 (1:1000; Pharmingen, San Diego, CA, USA), NR2A (1:1000; Zymed, San Francisco, CA, USA), NR2B (1:1000; Zymed), GluR1 (1:1500; Chemicon, Temecula, CA, USA), PSD-95 (1:2000; Affinity BioReagents, Golden, CO, USA), SAP97 (1:1000; StressGen, San Diego, CA, USA), Ca^2+^/calmodulin-dependent protein kinase II (αCaMKII; 1:3000; Chemicon), and p286-αCaMKII (1:1000; Promega, San Luis Obispo, CA, USA) in 3% albumin in TBS. After extensive rinsing in TBS/0.1% Tween 20, the nitrocellulose papers were incubated with horseradish peroxidase–conjugated secondary antibodies. Finally, the antigen–antibody complex was revealed by enhanced chemiluminescence (ECL; Amersham Biosciences, Little Chalfont, UK). Quantification was performed by means of a Quantity-One computer-assisted imaging system (Bio-Rad, Hercules, CA, USA).

### Intracellular Ca^2+^ imaging

We investigated acute effects of BDE-47 *in vitro* in PC12 cells. PC12 cells were subcultured in poly-l-lysine–coated glass-bottom cell culture dishes (MatTek, Ashland MA, USA) at 37°C, 5% CO_2_ as described previously (Westerink et al. 2000). We used the high-affinity Ca^2+^-responsive fluorescent dye Fura 2-AM (Molecular Probes; Invitrogen, Breda, the Netherlands) to measure the intracellular Ca^2+^ concentration. PC12 cells were incubated with Fura 2-AM (5 μM, 20 min at room temperature) in saline containing CaCl_2_ (1.8 mM), glucose (24 mM), Hepes (10 mM), KCl (5.5 mM), MgCl_2_ (0.8 mM), NaCl (125 mM), and sucrose (36.5 mM) at pH 7.3 (adjusted with NaOH). After incubation, the cells were washed with saline and left at room temperature for 15 min to allow intracellular deesterification of Fura 2-AM. After deesterification, the cells were placed on the stage of an Axiovert 35M inverted microscope (Zeiss, Göttingen, Germany) equipped with a TILL Photonics Polychrome IV (TILL Photonics GmBH, Gräfelfing, Germany). Fluorescence evoked by 340 and 380 nm excitation wavelengths (F340 and F380) was collected at 510 nm with an Image SensiCam digital camera (TILL Photonics GmBH). The digital camera and polychromator were controlled by imaging software (TILLvisION, version 4.01), which was also used for data collection and processing. The F340/F380 ratio, which is a qualitative measure for intra-cellular Ca^2+^ concentration, was measured every 20 sec during baseline. After 5 min baseline recording, BDE-47 was bath-applied to obtain final concentrations of 2 and 20 μM, and ratios were collected every 6 sec. Maximum and minimum ratios were determined after 25 min recording by addition of ionomycin (5 μM) and EDTA (17 mM) as a control for experimental conditions.

### Amperometry

We measured spontaneous and K^+^-evoked catecholamine release using carbon fiber microelectrode amperometry from isolated chromaffin cells and PC12 cells as described previously (Westerink and Vijverberg 2002). Chromaffin cells from mice exposed to vehicle or 68 mg (140 μmol)/kg bw BDE-47 were isolated and cultured as described previously (Westerink et al. 2006).

PC12 cells were superfused with BDE-47 for 15 min to investigate acute effects on vesicular catecholamine release. Recordings were performed at room temperature. PC12 cells with high basal release (> 5 events/min) or low evoked release (< 16 events/min) were excluded for data analysis (3/25 cells).

### Statistical analysis

All data are presented as mean ± SE. PC12 data were compared using Student’s paired *t*-test. We first compared the LTP data using a two-way analysis of variance (ANOVA) with *post hoc* Bonferroni testing (Sigmastat software; Systat Software Inc, Erkrath, Germany), followed by additional unpaired *t*-tests to specify the effects on PTP and LTP. We used unpaired Students’ *t*-test for all other data.

## Results

Pups exposed to BDE-47 did not differ in body weight and relative thymus weight compared with their unexposed littermates (data not shown), indicating the absence of general toxicity, treatment-dependent food competition, extensive immune suppression, and stress. Additionally, visual inspection of the brain slices of exposed pups did not show any changes of general hippocampus morphology (data not shown).

**Figure 2:**
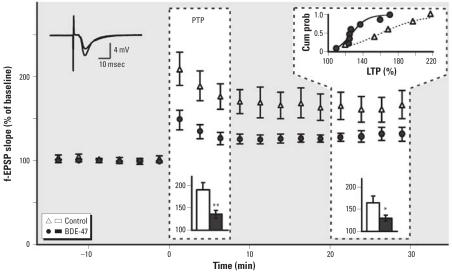
Exposure to 6.8 mg (14 μmol)/kg bw BDE-47 reduces PTP (0–7.5 min after tetanus at *t* = 0) and LTP (20–30 min) in hippocampal neurons in BDE-47–exposed mice (*n* = 8) compared with control mice (*n* = 5). The upper left inset shows superimposed traces illustrating the enhancement of the f-EPSP by LTP induction. The bar diagram insets in the dashed frames show averages of data. The upper right inset shows the cumulative probability (cum prob) curve of LTP in the individual experiments. *p < 0.05; **p < 0.01.

Figure 2 shows the results from f-EPSP recordings in the CA1 region of mouse hippocampus for control and BDE-47–exposed groups. No differences in stimulus–response relation were seen. No effects were observed on half-maximum f-EPSP slopes before LTP induction (control: 682 ± 138 V/sec; BDE-47 exposed animals: 679 ± 92 V/sec).

After tetanic stimulation, an immediate large increase of the f-EPSP is apparent, although the increase is significantly lower in the BDE-47–exposed group than in the control group. The increase of the f-EPSP during the first 7.5 min post-tetanus is classified as PTP. In the BDE-47–exposed mice, there was significantly less PTP (135 ± 9%) than in the control mice (190 ± 17%) (*p* < 0.01) (Figure 2). After PTP the f-EPSP size decreases but stabilizes at a higher level than baseline. This level of LTP is maintained for at least 30 min. In the BDE-47–exposed mice, LTP was significantly lower (130 ± 7%) than in the control group (165 ± 16%) (*p* < 0.05). The significance of these findings was confirmed by two-way ANOVA with *post hoc* Bonferroni testing. The trace inset illustrates the enhancement of f-EPSPs after tetanic stimulation. The cumulative probability curve of LTP in the individual experiments (Figure 2) indicates a shift to lower LTP values in the BDE-47 group.

**Figure 3:**
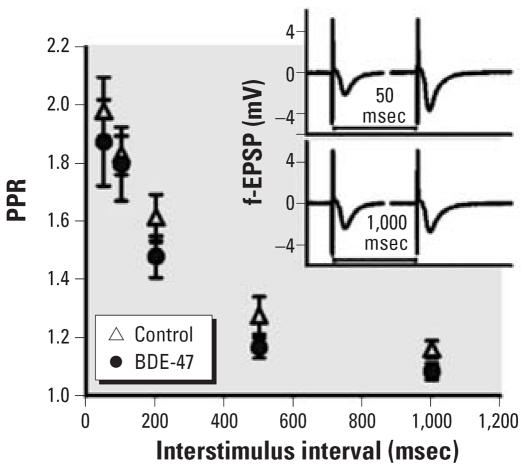
Exposure to 6.8 mg (14 μmol)/kg bw BDE-47 has no effects on PPF; PPR was calculated from paired pulses (interstimulus interval 50–1,000 msec) in control (*n* = 12) and BDE-47–exposed (*n* = 12) mice. Inset shows representative f-EPSP traces of paired pulses.

Figure 3 shows the effect of BDE-47 on PPF at different interstimulus intervals. For the 50-msec interstimulus interval, the PPR was 1.98 ± 0.11% in the control group and 1.87 ± 0.15% in the BDE-47 group. For the 1,000-msec interstimulus interval, the PPR was decreased to 1.16 ± 0.03% in the control group and 1.08 ± 0.03% in the BDE-47 group. Insets show representative recordings of PPF. No effects of BDE-47 on PPR were detected.

**Figure 4:**
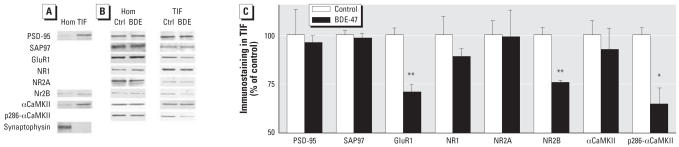
Effects of 6.8 mg (14 μmol)/kg bw BDE-47 on levels of postsynaptic proteins in the hippocampus of control (Ctrl; *n* = 4) and BDE-47–exposed (*n* = 4) mice. (*A*) Western blotting for NR2B, PSD-95, αCaMKII, and synaptophysin in homogenate (Hom) and TIF from hippocampus. (*B*) Representative Western blots of the investigated postsynaptic proteins in hippocampal homogenate (Hom) and TIF. (*C*) Relative amount of postsynaptic proteins in hippocampal TIF (representing the PSD). *p < 0.05. **p < 0.01.

Because activation of NMDA receptors is required for LTP, the reduction of LTP in BDE-47–treated mice could reflect an alteration of NMDA receptor-associated signaling elements. Because the NMDA receptor complex is enriched in the PSD, we used Western blot analysis to measure protein levels of NMDA receptor subunits and other PSD-associated signaling proteins in total homogenate and TIF, representing the PSD compartment by Western blot analysis (Gardoni et al. 2001). Protein composition of this preparation was carefully tested for the absence of presynaptic markers and enrichment in PSD proteins (Figure 4A; Gardoni et al. 2001). Representative Western blots for all investigated proteins in hippocampal homogenate and TIF are also shown in Figure 4B. BDE-47 had no effects on protein levels in cortical homogenate and TIF (data not shown) and hippocampal homogenate (Figure 4C). Figure 4C shows amounts of the proteins in TIF of the hippocampus of BDE-47–exposed mice compared with control mice. Significant changes in protein levels of NMDA receptor subunits NR1 and NR2A, and NMDA receptor interacting proteins PSD-95 and SAP97 were not detected. However, protein levels of NMDA receptor subunit NR2B (75 ± 2%) and α-amino-5-hydroxy-3-methyl-4-isoxazole propionic acid (AMPA) receptor subunit GluR1 (71 ± 4%) were significantly reduced (*p* < 0.01). There was a significant decrease in the autophosphorylated-active form of αCaMKII (p286-αCaMKII) to 65 ± 8% of control level (*p* < 0.05), although total αCaMKII was not changed.

In the experiments described above, post-synaptic effects of BDE-47 are observed, whereas presynaptic functional effects are not detected. However, possible effects on pre-synaptic mechanisms might remain undetected at a dose of 6.8 mg (14 μmol)/kg bw BDE-47. To ascertain the apparent absence of presynaptic effects of BDE-47, we investigated effects on catecholamine release in chromaffin cells obtained from mice exposed to vehicle or to a higher dose (68 mg (140 μmol)/kg bw) of BDE-47. No changes were detected in the different parameters of vesicular catecholamine release; that is, basal and high-K^+^ evoked release frequency and vesicular release parameters like quantal size (vesicle content), spike amplitude, and 50–90% rise time (data not shown).

**Figure 5:**
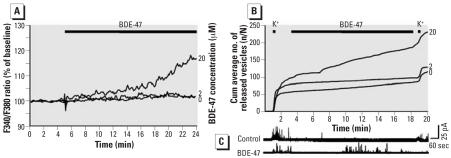
Acute effects of BDE-47 on Ca^2+^ and vesicular catecholamine release in PC12 cells. (*A*) Intracellular free Ca^2+^ (normalized F340/F380) in cells exposed to DMSO (*n* = 79), 2 μM BDE-47 (*n* = 32), or 20 μM BDE-47 (*n* = 27); base level (*t* = 2–4 min) and effect (*t* = 12–14 min) differed significantly for 20 μM BDE-47. (*B*) Cumulative (Cum) average number of amperometrically recorded vesicles from PC12 cells exposed to DMSO (*n* = 9), 2 μM BDE-47 (*n* = 7), or 20 μM BDE-47 (*n* = 6). (*C*) Representative amperometric traces of PC12 cells expsoed to DMSO (control) or 20 μM BDE-47.

Additional *in vitro* experiments were performed in PC12 cells to investigate acute effects of BDE-47 exposure on calcium homeostasis and release mechanisms. Figure 5A shows the average F340/F380 ratio in PC12 cells during bath application of DMSO, 2 μM BDE-47, and 20 μM BDE-47 normalized to baseline (first 5 min). The higher concentration of BDE-47 induced an increase in normalized F340/F380 ratio (*t* = 12–24 min, *p* < 0.01). To investigate whether the increase in intra-cellular Ca^2+^ has functional consequences, vesicular catecholamine release was also investigated (Figure 5B). The average number of amperometrically recorded events of vesicular release amounted to 1.9 ± 0.7 events/min (*n* = 9) in control experiments. During superfusion with 20 μM BDE-47, the release frequency was enhanced to 6.0 ± 1.7 events/min (*n* = 6; *p* < 0.05), whereas superfusion with 2 μM BDE-47 caused no detectable effect (1.2 ± 0.5 events/min; *n* = 7). BDE-47 had no effect on release evoked by high-K^+^ depolarization of the cells. Differences in vesicular release parameters could not be detected (data not shown).

## Discussion

A broad spectrum of neurotoxicants (e.g., environmental pollutants such as metals, pesticides, and PCBs) has been shown to cause a reduction of habituation after neonatal exposure (Eriksson et al. 1990, 1991; Eriksson and Fredriksson 1991; Fredriksson et al. 1992). However, from the behavioral effects it is difficult to deduce information about underlying mechanisms.

In the present study, we found that neonatal exposure to BDE-47 causes developmental effects consisting of a reduction of PTP and LTP, as well as specific reductions of key post-synaptic proteins involved in glutamate receptor signaling. Presynaptic parameters were not affected *ex vivo. In vitro* experiments on PC12 cells show an increase in intracellular Ca^2+^ and spontaneous vesicular release, only at the highest concentration BDE-47 (20 μM). The combined results suggest that presynaptic changes do not directly contribute to the observed defect in synaptic plasticity.

The exposure to BDE-47 took place during a period of rapid brain growth, which in mice takes place during the first 3–4 weeks of life, reaching its peak around PND10 (Davison and Dobbing 1968). The multitude and complexity of processes during this rapid development makes the developing brain particularly vulnerable to the effects of xenobiotics, like the adverse effect of BDE-47 on spontaneous behavior and habituation (Eriksson et al. 2001). Interestingly, exposure to BDE-47 does not affect performance in the Morris water maze test (Eriksson et al. 2001), commonly used as a learning task to detect effects in the hippocampus. This suggests that habituation is a more sensitive parameter for BDE-47 effects in the hippocampus.

We observed a specific reduction of key proteins in the PSD (i.e., GluR1, NR2B, and p286-αCaMKII). Because no changes were observed in total hippocampus homogenate, the specific decrease in the PSD is therefore attributed to changes in glutamate receptor subunit trafficking or clustering in the PSD instead of a reduced protein translation.

A study in GluR1-knockout mice showed that approximately 10% of the normal amount of GluR1 is sufficient for LTP (Mack et al. 2001). Also, a GluR1-independent form of LTP has been observed in juvenile GluR1-knockout mice (Jensen et al. 2003). Therefore, major effects on LTP as a consequence of the observed reduction of AMPA subunit GluR1 by approximately 30% are not expected.

The observed reduction of NR2B subunits results in an increased NR2A/NR2B ratio. The majority of NMDA receptors consist of 2 NR1 and 2 NR2A or 2 NR2B subunits. NR2A-NMDA receptors gate smaller Ca^2+^ currents, have a lower affinity for glutamate, and desensitize faster than NR2B-NMDA receptors (Kutsuwada et al. 1992). Therefore, an increased NR2A/NR2B ratio is likely to result in a higher threshold for LTP induction, which could explain the reduction of PTP and LTP.

In mice exposed to BDE-47, the auto-phosphorylated-active form of αCaMKII was significantly reduced. Because CamKII autophosphorylation is essential for hippocampal NMDA-dependent LTP (Giese et al. 1998), this specific effect may lead to reduced synaptic plasticity resulting in behavioral impairments.

To ascertain the absence of presynaptic effects, we investigated neurotransmitter release from chromaffin cells from BDE-47–exposed [68 mg (140 μmol)/kg bw] mice. Because PPR and chromaffin neurotransmitter release remained unchanged after developmental exposure to BDE-47, and because modest acute effects on free intracellular Ca^2+^ and spontaneous vesicular catecholamine release in PC12 cells were only detected at a concentration of 20 μM BDE-47, we propose that presynaptic changes do not contribute considerably to the observed functional defect in synaptic plasticity. Based on tissue distribution data for 1 mg/kg bw ^14^C-BDE-47 orally given to C57Bl/6 mice on PND10 (Staskal et al. 2006), brain concentration at sacrifice after exposure to 6.8 mg (14 μmol)/kg bw BDE-47 is estimated to be 0.43–0.81 μM and the peak brain concentration, reached 8 hr after exposure, is estimated to be 1.1 μM. These estimated concentrations are at least one order of magnitude lower than the lowest effective concentration in the *in vitro* experiments described here.

As with PCBs (for review, see Fonnum et al. 2006), *in vitro* exposure to the commercial penta-BDE mixture DE-71, which contains (on a weight basis) 31.8% BDE-47 (Reistad and Mariussen 2005), affects several other transmitter systems. Previous studies reported cell death of cerebellar granule cells, alterations of Ca^2+^ homeostasis in human neutrophils and brain microsomes, and arachidonic acid release and protein kinase C translocation in cerebellar granule cells; inhibition of dopamine reuptake in rat brain synaptic vesicles has been reported after *in vitro* exposure to DE-71 in the micromolar range (2–20 μM) (Kodavanti and Ward 2005; Mariussen and Fonnum 2003; Reistad et al. 2002; Reistad and Mariussen 2005). Interestingly, addition of the NMDA receptor antagonist MK801 protects cerebellar granule cells against DE-71-induced cell death (Reistad et al. 2006). No other effects of PBDEs on glutamate receptors have yet been published.

Pure (~ 99%) BDE-47, which has been used in only a few experiments, has revealed formation of reactive oxygen species in human neutrophils and increased ^3^H-phorbol ester binding in primary rat cerebellar granule neurons, also at micromolar concentrations (Kodavanti et al. 2005; Reistad and Mariussen 2005). The effects of BDE-47 in PC12 cells reported here occur at concentrations in the same order of magnitude.

Effects on spontaneous motor activity and habituation in mice have been described for several lower and higher brominated diphenyl ethers after a single oral dose of maximally 21 μmol/kg bw (Branchi et al. 2002, 2003; Eriksson et al. 2001, 2002; Viberg et al. 2003a, 2003b, 2006). In rats, effects on behavior have been observed after maternal exposure to 10 mg (18 μmol)/kg bw BDE-99 at gestational days 10–18 and after oral exposure to 30 mg/kg bw DE-71 at PND6–12 (Dufault et al. 2005; Lilienthal et al. 2006).

In the 1990s, an association between delayed human neurodevelopment and pre-natal or early exposure to PCBs was reported by cohort studies. These results were corroborated by experiments demonstrating the developmental neurotoxicity of PCBs. The observed interaction with the thyroid hormone system is usually considered part of the underlying mechanism (for review, see Winneke et al. 2002). For hazard characterization of PCBs and the structurally related PBDEs, it is relevant to investigate whether they induce similar effects through similar mechanisms. This is of particular importance because, in neonatal mice, the effects of a combined dose of PCB-52 and BDE-99 on spontaneous motor behavior and habituation capability appear to be additive or perhaps even synergistic (Eriksson et al. 2006).

High human serum concentrations of BDE-47 were measured in female inhabitants of California by Petreas et al. (2003); the concentration of BDE-47 in serum ranged from 5 to 510 ng/g lipid weight, with a median of 16.5 ng/g lipid weight. High concentrations (> 100 ng/g lipid weight) have also been reported in Californian children (Fisher et al. 2006). The highest and median values correspond (using average physiologic values) to blood concentrations of approximately 11.5 nM and approximately 0.37 nM. Using the tissue distribution data for 1 mg/kg bw ^14^ C-BDE-47 (Staskal et al. 2006), the dose used in the current study corresponds to an estimated blood concentration of approximately 2.6 μM after 3 hr and to approximately 0.6 μM after 10 days (i.e., ~ 50–200 times higher than in the worst, and ~ 1,600–7,000 times higher than in the median human situation described above). For risk assessment, the difference between the animal dose level causing an adverse effect and the highest human dose levels is relatively small, considering safety factors for species extrapolation and intra-species variability. Additional uncertainty comes from the fact that humans are exposed to multiple flame retardants over a lifetime. Accumulation of BDE-47, as demonstrated in primary rat cerebellar granule neurons and primary rat neocortical cells (Kodavanti et al. 2005; Mundy et al. 2004), is another reason for concern about the neurotoxic potential of PBDEs.

No tolerable daily intake is assigned to PBDEs because sufficient data are not available. However, the limited toxicity data suggest that adverse effects induced by exposure to the more toxic congeners in rodents occur at doses of at least 100 μg/kg bw per day [Joint FAO/WHO Expert Committee on Food Additives (JECFA) 2005]. The combination of quantitative molecular data with functional neurophysiologic effects reported here provides strong functional support for the previously reported neurobehavioral effects (Eriksson et al. 2001) and is essential for characterization of the neurotoxic hazard of brominated flame retardants, particularly for rational risk assessment, which is required in response to the general concern about the vulnerability of the developing brain.
